# Associations Between Lifestyle Factors and Primary Dysmenorrhea in the Japan Nurses’ Health Study

**DOI:** 10.1177/26884844251362183

**Published:** 2025-07-23

**Authors:** Satoshi Obayashi, Yuki Ideno, Toshiro Kubota, Kiyoshi Takamatsu, Kunihiko Hayashi

**Affiliations:** ^1^ Department of Obstetrics and Gynecology, Dokkyo Medical University School of Medicine, Tochigi, Japan.; ^2^ Gunma University Center for Food Science and Wellness, Gunma, Japan.; ^3^ Gunma University Initiative for Advanced Research, Gunma, Japan.; ^4^ Tokyo Kyosai Hospital, Tokyo, Japan.; ^5^ Department of Obstetrics and Gynecology, Tokyo Dental College Ichikawa General Hospital, Chiba, Japan.; ^6^ Tsukubamirai Endo Ladies Clinic, Ibaraki, Japan.; ^7^ Graduate School of Health Data Science, Juntendo University, Chiba, Japan.

**Keywords:** alcohol consumption, menstrual symptom, physical activity, smoking, soybean intake, sleep duration

## Abstract

**Background:**

Dysmenorrhea is chronic and cyclic pain during menstruation and is a common gynecological problem worldwide. Although there are several reported risk factors for dysmenorrhea, the findings of previous studies are inconsistent. This study aimed to identify lifestyle factors associated with dysmenorrhea.

**Methods:**

The study population comprised 36,665 premenopausal female nurses aged 20–49 years who completed the Japan Nurses’ Health Study (JNHS) baseline survey. Out of grades 0–4 of menstrual pain, dysmenorrhea was defined as grades 2–4. Multivariable modified Poisson regression analysis was used to examine the associations between dysmenorrhea and possible risk factors, namely age, current menstrual cycle, marital status, parity, current body mass index (BMI), smoking status, alcohol consumption, work shift, physical activity, sleep duration, and soybean isoflavone intake.

**Results:**

There was a significant negative association between age and the prevalence ratio (PR) of dysmenorrhea (*p* < 0.0001). Older age and parity were significantly associated with decreased multivariable-adjusted PRs. The factors significantly associated with increased PRs were an irregular menstrual cycle, being underweight (BMI < 18.5 kg/m^2^), smoking, consuming alcohol, short sleep duration, and night shift work. Although the age-adjusted PRs of total isoflavone aglycone equivalents, tofu intake, and miso soup intake showed a significant linear trend toward decreased PRs, there was a significant decrease in multivariable-adjusted PRs only in the “almost every day” tofu-intake group.

**Conclusion:**

The JNHS baseline survey revealed that the factors associated with dysmenorrhea in Japanese women were age, parity, menstrual cycle, being underweight, and lifestyle factors, including soybean food intake.

## Introduction

Dysmenorrhea is defined as chronic and cyclic pain during menstruation and is still one of the most common gynecological problems worldwide.^1^ It is well known that the degree and severity of dysmenorrhea are influenced by community, lifestyle, religion, work, and education statuses.^2^ As menstrual pain decreases quality of life (QOL), it is very important to properly assess dysmenorrhea to improve the QOL of affected women.

In the United States, over two million females experience dysmenorrhea, resulting in appointments with medical doctors, missed school/work, or bed rest.^3^ The estimated prevalence of menstrual pain is 72% in Sweden,^4^ 33% in India,^5^ and 51% in Singapore.^6^ A recent study shows that 10% of females are absent from work for 1–3 days per month because of menstrual pain and are not capable to carry out their normal daily activities.^7^ Furthermore, menstrual pain is the most important cause of student absence from school, disadvantaging females during their school life.^8^ The estimated annual economic burden as work productivity loss due to menstrual symptoms in the Japanese female population was reportedly up to 683 billion Japanese yen (8.6 billion USD) in 2017.^9^

Several risk factors for menstrual pain have been reported, but the results of previous studies are heterogeneous. Some studies have showed that pregnancy is a protective factor that reduces the severity of dysmenorrhea,^10,11^ whereas others have showed that the severity of dysmenorrhea is related to a short menstrual cycle but is not related to parity.^12^

Soybeans contain isoflavones that are metabolized by some intestinal bacteria into equol, which is one of the metabolites of daidzein and has a strong affinity for estrogen receptors. It has been suggested that isoflavones reduce estrogen activity through prostaglandin E2 (PGE2) production and thus reduce dysmenorrhea.^13^ Furthermore, equol is known to reduce the symptoms of premenstrual syndrome, including abdominal pain or irritation,^14^ although this has not been clarified in an epidemiological study.

The objectives of the present study were to evaluate the prevalence of dysmenorrhea in Japanese women who completed the Japan Nurses’ Health Study (JNHS) and to compare the influence of reproductive and lifestyle factors, such as smoking status, alcohol consumption, sleep duration, physical activity, and isoflavone intake, on the severity of dysmenorrhea.

## Materials and Methods

### Study design and study population

The JNHS is a large-scale, nationwide prospective cohort study investigating the effects of lifestyle habits and health care practices on Japanese women’s health throughout their life. The objectives of the JNHS are to prospectively monitor the occurrence of various diseases and states of health, including female-specific diseases such as dysmenorrhea, and to assess the effects of various lifestyle factors and nutritional habits on the health of Japanese women. The JNHS consists of a cross-sectional baseline survey and a longitudinal follow-up survey. The cross-sectional baseline survey was conducted between 2001 and 2007 and was completed by a total of 49,927 female nurses throughout Japan. Among these women, the present study selected premenopausal female nurses aged 20–49 years (*n* = 37,798). After the exclusion of pregnant women (*n* = 983) and nonpregnant women who did not report their degree of dysmenorrhea (*n* = 150), the present study cohort comprised 36,665 nonpregnant and premenopausal female nurses who had regular or irregular menstruation.

Basic information on medical, anthropometric, reproductive, and dietary factors, including body weight, cigarette smoking status, alcohol consumption, parity, age at menarche, menopausal status, and whether the individual worked night shift, was collected *via* a self-administered questionnaire administered as the baseline survey.^15^

This project was conducted in accordance with the international guidelines of Good Epidemiology Practice and the Japanese Ethical Guidelines for Epidemiological Research. The study design and study protocol were reviewed and approved by the Gunma University Hospital Clinical Research Review Board (#101, July 30, 2001). Participants were informed about the purposes and procedures of this study in an invitation letter, and all of the signed consent forms were sent to the JNHS coordination center.

### Dysmenorrhea

To evaluate the prevalence and severity of dysmenorrhea, the symptoms were classified into five grades in the questionnaire. Grade 0: no pain during menstruation; grade 1: slight menstrual pain, but able to carry on daily life during menstruation; grade 2: moderate menstrual pain, but able to carry on daily life with utilizing analgesics; grade 3: severe menstrual pain that affected daily life and imposed staying home with taking analgesics; grade 4: very severe menstrual pain with having difficulty in walking and requiring bed rest all day. Women who had grades 2, 3, and 4 dysmenorrhea were categorized as the dysmenorrhea group, whereas women with grades 0 and 1 were categorized as the nondysmenorrhea group.

### Reproductive and lifestyle factors

The current menstrual cycle was asked in six categories: ≤21 days, 21–25 days, 26–31 days, 32–39 days, 40–50 days, or >50 days or too irregular,^16^ which was then recategorized into four categories: ≤25 days, 26–31 days, 32–50 days, and irregular. The marital status was categorized as single or married (including divorced and widowed). Parity was categorized as “none” and “one or more.” Current body mass index (BMI) was calculated as the weight (kg)/height (m^2^) and was categorized as <18.5 kg/m^2^, 18.5 to <25.0 kg/m^2^, 25.0 to <30.0 kg/m^2^, and ≥30.0 kg/m^2^.

The smoking status was classified as “never smoked,” “current smoker,” “ex-smoker <5 years since quitting,” “ex-smoker ≥5 years since quitting,” and “ex-smoker unknown duration since quitting.” The frequency of alcohol consumption was categorized as “none,” “1 or 2 days/week,” and “3 or more days/week.” Moderate-to-vigorous intensity physical activity was defined as physical activity for over 10 MET hours per week and was categorized as “yes” or “no.” In the present study, the participants’ physical activity levels were assessed using a previously reported method.^17^ Sleep duration was categorized as <5 hours per day, ≥9 hours per week, and unknown.

The frequency of consumption of the soybean-containing food products tofu, miso soup, and natto was categorized into five categories as follows: never, once a week, 2–3 days per week, 4–5 days per week, and almost every day. The total isoflavone intake as aglycone equivalents (mg/week) for each participant was estimated using the intake frequencies for tofu, miso soup, and natto in the baseline survey; median quantities per serving in the 98-item semiquantitative food-frequency questionnaires in the 6-year follow-up survey,^18^ and soy isoflavone concentration as aglycone forms in the Japanese Standard Tables of Food Composition, enlarged fifth edition.^19^ The total isoflavone intake was then grouped into quintiles according to the no. of participants.

### Statistical analysis

The prevalence of the five grades of dysmenorrhea was estimated in the following five age groups: 20–29 years, 30–34 years, 35–39 years, 40–44 years, and 45–49 years. The Cochran–Armitage trend test was used to compare the prevalence of dysmenorrhea (grades 2, 3, and 4) among the age groups.

Multivariable modified Poisson regression analysis with the robust sandwich variance was used to estimate the age-adjusted prevalence ratios (PRs) and their 95% confidence intervals (CIs) for each risk factor independently. Multivariable modified Poisson regression analysis was also used to examine the associations of potential risk factors (age group, current menstrual cycle, marital status, parity, current BMI, smoking status, alcohol consumption, working the night shift, physical activity, sleep duration, and total soybean isoflavone intake) with dysmenorrhea. We divided participants into quintiles of total soybean isoflavone intake (mg/week) as Q1: <66.7, Q2: 66.7 to <103.5, Q3: 103.5 to <145.2, Q4: 145.2 to <205.5, and Q5: ≥205.5, and used the lowest quintile group as the reference. The associations between the intake of soybean food products (tofu, miso soup, and natto) and dysmenorrhea were examined.

All statistical data analyses were carried out using SAS version 9.4 (SAS Institute, Cary, NC, USA). *p* < 0.05 was regarded as statistically significant.

## Results

### Study population characteristics

The total no. of participants was 36,665, and the demographics and lifestyle characteristics of the study population are shown in Table 1. The mean age of the participants was 38.4 ± 5.7 years, and the average current BMI was 21.6 ± 3.0 kg/m^2^. Among the total cohort, 68.6% had never smoked (*n* = 25,181), 18.6% were current smokers (*n* = 6,830), and 11.6% were ex-smokers (*n* = 4,227).

**Table 1.** Distribution of Demographic and Lifestyle Variables in the Study Population (*n* = 36,665)

	Mean ± SD	*n*	%
Age (years)	38.4 ± 5.7		
20–29		871	2.4
30–34		10,381	28.3
35–39		9,681	26.4
40–44		9,113	24.9
45–49		6,619	18.1
Current menstrual cycle			
≤25 days		4,314	11.8
26–31 days		22,155	60.4
32–50 days		3,512	9.6
Irregular		6,414	17.5
Unknown		270	0.7
Marital status			
Single		10,246	27.9
Married^a^		26,060	71.1
Unknown		359	1.0
Parity			
None		12,443	33.9
One or more		22,880	62.4
Unknown		1,342	3.7
BMI (kg/m^2^)	21.6 ± 3.0		
<18.5		3,821	10.4
18.5 to <25.0		27,778	75.8
25.0 to <30.0		3,423	9.3
≥30.0		653	1.8
Unknown		990	2.7
Smoking status			
Never smoked		25,181	68.6
Current smoker		6,830	18.6
Ex-smoker: <5 years since quitting		1,527	4.2
Ex-smoker: ≥5 years since quitting		2,550	7.0
Ex-smoker: unknown duration since quitting		150	0.4
Unknown		427	1.2
Alcohol consumption			
None		10,150	27.7
≤2 days per week		16,142	44.0
≥3 days per week		8,386	22.9
Unknown		1,987	5.4
Engaging in night duty			
Yes		30,140	82.2
No		5,888	16.1
Unknown		637	1.7
Moderate-to-vigorous physical activity^b^ (≥10 MET hours/week)			
Yes		1,481	4.0
No		24,043	65.6
Unknown		11,141	30.4
Sleep duration			
<5 hours/day		740	2.0
5 hours/day		5,231	14.3
6 hours/day		15,382	42.0
7 hours/day		9,499	25.9
8 hours/day		3,892	10.6
≥9 hours/day		374	1.0
Unknown		1,547	4.2
Total isoflavone intake: aglycone equivalents (mg/week)	145.7 ± 94.5		
Q1: ≤66.7		7,133	19.5
Q2: 66.7 to <103.5		5,867	16.0
Q3: 103.5 to <145.2		8,322	22.6
Q4: 145.2 to <205.5		7,545	20.6
Q5: ≥205.5		7,288	19.9
Unknown		510	1.4

^a^ Includes divorced and widowed.

^b^ Moderate-to-vigorous physical activities: intensity ≥3 METs.

BMI, body mass index.

### Prevalence of dysmenorrhea by age group

**FIG. 1.**
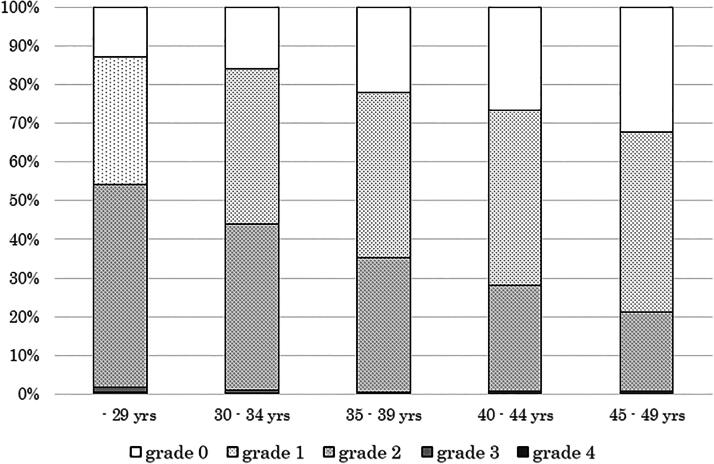
Prevalence of dysmenorrhea by age group. Grade 0: no pain during menstruation; grade 1: slight menstrual pain, but able to carry on daily life during menstruation; grade 2: moderate menstrual pain, but able to carry on daily life with utilizing analgesics; grade 3: severe menstrual pain that affected daily life and imposed staying home with taking analgesics; and grade 4: very severe menstrual pain having difficulty in walking and required bed rest all day. Cochran–Armitage trend test for the prevalence of dysmenorrhea (grades 2, 3, and 4): *p* < 0.0001.

Figure 1 indicates the prevalence and severity of dysmenorrhea among the five age groups. The prevalence of dysmenorrhea (grades 2, 3, and 4) was 54.1% in the women aged 20–29 years group, 44.0% in women aged 30–34 years, 35.3% in women aged 35–39 years, 28.1% in women aged 40–44 years, and 21.1% in women aged 45–49 years. There was a significant negative association between age and the prevalence of dysmenorrhea (Cochran–Armitage trend test; *p* < 0.0001).

### Association between dysmenorrhea and menstrual status/lifestyle factors

The age-adjusted PRs and multivariable-adjusted PRs were estimated to examine the associations between possible risk factors and the prevalence of dysmenorrhea (grades 2, 3, and 4) (Table 2). A sensitivity analysis performed by recalculating the PRs after excluding the “unknown” group provided similar results (data not shown). The factors significantly associated with a decreased prevalence of dysmenorrhea were older age and a parity of one or more. The factors significantly associated with an increased prevalence of dysmenorrhea were an irregular menstrual cycle, being underweight (BMI <18.5 kg/m^2^), smoking, alcohol consumption, and night shift work.

**Table 2.** Prevalence Ratios of Reproductive and Lifestyle Factors

	Age-adjusted PR		Multivariable-adjusted PR^a^	
	PR	95% CI	PR	95% CI
Age (years)				
20–29			Ref	
30–34			0.90	0.84–0.96
35–39			0.79	0.74–0.85
40–44			0.67	0.62–0.72
45–49			0.51	0.47–0.55
Current menstrual cycle				
≤25 days	1.01	0.96–1.06	0.99	0.95–1.05
26–31 days	Ref		Ref	
32–50 days	0.97	0.92–1.01	0.97	0.93–1.02
Irregular	1.17	1.13–1.21	1.14	1.10–1.18
Unknown	0.91	0.76–1.10	0.91	0.76–1.09
Marital status				
Single	Ref		Ref	
Married^b^	0.77	0.74–0.79	1.03	0.99–1.08
Unknown	0.80	0.69–0.93	0.87	0.74–1.03
Parity				
None	Ref		Ref	
One or more	0.69	0.67–0.71	0.70	0.67–0.73
Unknown	0.94	0.88–1.01	0.94	0.88–1.01
BMI				
<18.5	1.14	1.10–1.19	1.11	1.06–1.15
18.5 to <25.0	Ref		Ref	
25.0 to <30.0	1.02	0.97–1.08	1.01	0.96–1.07
≥30.0	1.06	0.95–1.18	0.99	0.89–1.11
Unknown	1.08	0.99–1.17	1.06	0.97–1.16
Smoking status				
Never smoked	Ref		Ref	
Current smoker	1.27	1.23–1.32	1.20	1.16–1.25
Ex-smoker: <5 years since quitting	1.14	1.07–1.22	1.15	1.08–1.23
Ex-smoker: ≥5 years since quitting	1.14	1.08–1.20	1.12	1.06–1.18
Ex-smoker: unknown duration	1.06	0.85–1.33	1.05	0.84–1.32
Unknown	1.03	0.89–1.19	1.03	0.88–1.20
Alcohol consumption				
None	Ref		Ref	
≤2 days per week	1.10	1.06–1.14	1.05	1.01–1.08
≥3 days per week	1.19	1.15–1.24	1.12	1.07–1.17
Unknown	1.04	0.97–1.12	0.99	0.93–1.07
Engaging in night shift				
No	Ref		Ref	
Yes	1.12	1.07–1.17	1.06	1.01–1.10
Unknown	1.07	0.95–1.21	1.02	0.89–1.16
Moderate-to-vigorous physical activity^c^ (≥10 MET hours/week)				
No	Ref		Ref	
Yes	1.13	1.06–1.21	1.10	1.03–1.17
Unknown	1.02	0.99–1.05	0.99	0.97–1.03
Sleep duration				
<5 hours/day	1.46	1.34–1.58	1.35	1.25–1.47
5 hours/day	1.25	1.19–1.30	1.19	1.14–1.25
6 hours/day	1.12	1.08–1.16	1.09	1.05–1.13
7 hours/day	Ref		Ref	
8 hours/day	0.93	0.88–0.99	0.95	0.90–1.01
≥9 hours/day	1.09	0.96–1.25	1.09	0.95–1.25
Unknown	1.10	1.02–1.19	1.06	0.98–1.15
Total isoflavone aglycone equivalents (mg/week)				
Q1	Ref		Ref	
Q2	0.94	0.90–0.99	1.00	0.96–1.05
Q3	0.92	0.88–0.96	1.00	0.96–1.05
Q4	0.91	0.87–0.95	1.01	0.97–1.05
Q5	0.88	0.84–0.92	0.97	0.92–1.01

^a^ Adjusted for age, current menstrual cycle, parity, marital status, BMI, smoking status, alcohol consumption, engaging in night shift, physical activity, sleep duration, and isoflavone intake.

^b^ Includes divorced and widowed.

^c^ Moderate-to-vigorous physical activity: intensity ≥3 METs.CI, confidence interval; PR, prevalence ratio.

Current smokers were associated with a 20% increase in the risk of dysmenorrhea compared with those who had never smoked. Furthermore, the risk of dysmenorrhea slightly decreased after quitting smoking but was not affected by the duration of smoking cessation (over or under 5 years). For the total isoflavone aglycone equivalents, although the age-adjusted PRs were significantly decreased in Q2–Q5 compared with Q1, there were no significant decreases in multivariable-adjusted PRs.

### Soybean intake and reduction of dysmenorrhea

The age-adjusted PRs in Table 3 indicate a linear trend between the frequency of intake of the three kinds of soybean (tofu, miso soup, and natto) and the prevalence of dysmenorrhea. Multivariable-adjusted analysis showed that the intake of tofu “almost every day” significantly reduced the prevalence of dysmenorrhea. Therefore, tofu intake was shown to reduce dysmenorrhea in a dose-dependent manner. In contrast, multivariable-adjusted analysis showed no significant associations between the intakes of miso soup and natto and the prevalence of dysmenorrhea. A sensitivity analysis performed by recalculating the PRs after excluding the “unknown” group provided similar results (Supplementary Tables S1 and S2).

**Table 3.** Dysmenorrhea and the Frequency of Soybean Consumption

	Dysmenorrhea				Trend test^b^	Age-adjusted PR			Multivariable-adjusted PR^c^		
	Yes^a^		No								
	*n*	%	*n*	%		PR	95% CI	Linear trend	PR	95% CI	Linear trend
Tofu					*p* < 0.0001			*p* < 0.0001			*p* = 0.0118
Never	774	41.1	1,109	58.9		Ref			Ref		
Once a week	4,056	36.9	6,937	63.1		0.93	0.88–0.98		0.98	0.92–1.04	
2–3 days a week	4,661	33.1	9,433	66.9		0.87	0.82–0.92		0.96	0.91–1.03	
4–5 days a week	1,683	30.5	3,839	69.5		0.83	0.77–0.88		0.94	0.88–1.01	
Almost every day	1,049	28.6	2,614	71.4		0.80	0.74–0.86		0.92	0.85–0.99	
Unknown	187	36.7	323	63.3							
Miso soup					*p* < 0.0001			*p* < 0.0001			*p* = 0.7092
Never	941	40.8	1,365	59.2		Ref			Ref		
Once a week	2,028	37.3	3,405	62.7		0.94	0.88–0.99		0.98	0.93–1.04	
2–3 days a week	3,458	34.8	6,480	65.2		0.90	0.85–0.95		0.99	0.94–1.05	
4–5 days a week	2,121	32.8	4,337	67.2		0.86	0.81–0.91		0.99	0.94–1.06	
Almost every day	3,675	30.6	8,345	69.4		0.83	0.79–0.88		0.99	0.94–1.06	
Unknown	187	36.7	323	63.3							
Natto					*p* = 0.0144			*p* = 0.1737			*p* = 0.5385
Never	3,346	34.4	6,394	65.6		Ref			Ref		
Once a week	4,354	33.8	8,520	66.2		0.98	0.95–1.02		1.00	0.97–1.04	
2–3 days a week	2,977	34.3	5,698	65.7		1.00	0.97–1.04		1.05	1.00–1.09	
4–5 days a week	874	31.6	1,889	68.4		0.93	0.88–0.99		0.97	0.91–1.03	
Almost every day	672	32.0	1,431	68.0		0.97	0.91–1.04		1.01	0.94–1.08	
Unknown	187	36.7	323	63.3							

^a^ Yes: severity of dysmenorrhea is grades 2, 3, and 4.

^b^ Cochran–Armitage trend test.

^c^ Adjusted for age, current menstrual cycle, parity, marital status, BMI, smoking status, alcohol consumption, engaging in night shift, physical activity, and sleep duration.

## Discussion

Dysmenorrhea reportedly has a significant impact on education, and 20% of young women are absent from school during menstruation.^8^ Dysmenorrhea also decreases the QOL of working women. Therefore, it is very important to analyze the factors associated with dysmenorrhea to improve women’s QOL.

It is well known that primary dysmenorrhea occurs only in ovulatory cycles,^20^ indicating that adequate uterine exposure to estrogen and progesterone is necessary to express the symptoms. Furthermore, menarche occurs later in puberty, and there are increases in smoking and drinking behavior during the pubertal years.^21^ Therefore, it is crucial to examine the association between these lifestyle factors and menstrual symptoms when evaluating the etiology of menstrual problems. The present study found that smoking and alcohol consumption were factors associated with dysmenorrhea, whereas having an irregular menstrual cycle indicated a decreased PR of dysmenorrhea, which might suggest insufficient secretion of female hormones.

The present study found negative associations between the prevalence of dysmenorrhea and age and parity. A previous questionnaire-based study that analyzed the associations between dysmenorrhea and age and parity in 3,941 Japanese women who usually required analgesics found that the age-adjusted prevalence of dysmenorrhea decreases with increasing age and parity.^22^ This previous study reported that the prevalence of dysmenorrhea among women aged 25–29 years with a parity of zero was about 55%,^21^ which is very close to the prevalence of dysmenorrhea (grades 2, 3, and 4) in our study (54.1%) among women aged 20–29 years with or without parity. The similarity of these data suggests that the value might be the actual average prevalence of dysmenorrhea among young Japanese women.

A recent meta-analysis of 27,091 participants in 24 studies revealed that smokers are 1.45 times more likely to develop dysmenorrhea than nonsmokers (95% CI: 1.30–1.61)^23^ because the nicotinic action in cigarettes results in dysmenorrhea. In addition, other studies have indicated that the nicotine in cigarettes may cause vasoconstriction, resulting in hypoxia that causes myometrial contraction.^24^ This vasoconstriction leads to dysmenorrhea by decreasing the endometrial blood flow, which is common in women with dysmenorrhea. Therefore, quitting smoking should reduce dysmenorrhea;^25^ however, the present study found that quitting smoking resulted in minimal reduction of dysmenorrhea. The multivariable-adjusted PRs showed a 12% increase in dysmenorrhea in women who quit smoking more than 5 years ago compared with that in nonsmokers and an 8% decrease compared with that in current smokers (Table 2). These findings may suggest that a longer period may be required to eliminate or diminish the effects of smoking on dysmenorrhea in the present study population or may suggest the existence of some other mechanism (*e.g.,* physical or psychological factors) that affects the occurrence of dysmenorrhea at 5 years after the cessation of smoking.

Wang et al. reported that perceived stressful menstruation cycles have an independent effect on the occurrence of dysmenorrhea in Chinese women,^26^ as stress might regulate female hormonal conditions, resulting in altered progesterone synthesis. We found that nurses who worked the night shift had a 6% increase in the prevalence of dysmenorrhea, which might be partially explained by the creation of neuroendocrine responses expressed during night duty to increase prostaglandins (PGs), resulting in uterine contraction.^27^ Therefore, such working stress might have an influence on dysmenorrhea after the cessation of smoking, and these hormonal changes might be related to the remaining presence of dysmenorrhea in nurses who are ex-smokers. However, this issue requires further investigation in future research.

A review of the factors associated with the prevalence of dysmenorrhea indicated that a low BMI, related to low available energy in female athletes, results in the disruption of the hypothalamus–pituitary system, and this abnormal physical energy status leads to ovarian dysfunction, which induces insufficient secretion of female hormones.^28^ Therefore, the authors proposed that maintaining an appropriate BMI is important to reduce severe dysmenorrhea.^28^ In the present study, a low BMI (<18.5 kg/m^2^) increased the PR of dysmenorrhea by 11%. The mechanism may be the low secretion of female hormones in women with a low BMI.

The same review suggested that increased stress stimulates adrenaline and cortisol production, resulting in increased PG secretion and an increase in dysmenorrhea.^28^ Furthermore, a short sleep duration leads to decreased melatonin secretion, which is related to the pituitary–gonadal function and causes dysmenorrhea.^28^ The factors of “engaging in night duty” and “sleep duration under 6 hours/day” may have increased the PR of dysmenorrhea for similar reasons. However, alternatively, dysmenorrhea might induce a short sleep duration. The present study findings are not able to identify the causal relationship between these two factors.

Several local and molecular mechanisms around the uterus that cause primary dysmenorrhea have been reported.^27^ Previous studies have found the increased PG levels in the endometrium and menstrual blood during dysmenorrhea, and therefore, these increased PGs are now the strongest candidates to cause dysmenorrhea.^20^ Both PGF2α and PGE2 are known to be chemical stimulators that make myometrial and vascular contractions, which induce uterine hypoxia, resulting in lower abdominal pain as a symptom of dysmenorrhea.

Isoflavones are phytoestrogens found mainly in soybeans that inhibit PGE2 production^13^ and cyclooxygenase activity.^29^ In addition, equol, one of the most active metabolites of isoflavone, is also related to the reduction of dysmenorrhea.^30^ Therefore, the habitual intake of soybean may have a beneficial effect on decreasing the symptoms of dysmenorrhea by inhibiting cyclooxygenase activity and reducing the release of PGs. Although only 41.5% of Japanese women produce equol,^31^ we did not evaluate the equol producibility of each participant in the present study. This may lessen the strength of the association between soybean intake and dysmenorrhea. Our data show that the frequency of taking tofu containing soybean-derived isoflavone was negatively associated with the prevalence of dysmenorrhea. Considering the mechanism of dysmenorrhea as abnormal myometrial contraction, these data suggest that eating the soybean in tofu may reduce the uterine and vascular smooth muscle contractions. However, the intake of miso soup and natto (which also contain isoflavone) did not show a significant negative relationship with dysmenorrhea. Although the precise mechanism of the difference between eating tofu versus miso soup/natto was not clarified, these three products might contain different amounts of isoflavones or have different absorption rates. The estimated intake of the total isoflavone content as aglycone equivalents (*i.e.*, the glycoside-replaced form of isoflavone) was calculated using the individual habitual data, and there was a tendency for an inverse relationship between aglycone intake and the prevalence of dysmenorrhea (Table 2), which might partly support the beneficial effect of isoflavone in preventing dysmenorrhea. However, this issue requires further investigation.

The present study has some limitations. First, our study population consisted of female nurses who were relatively homogeneous regarding socioeconomic factors and likely to have different risk and lifestyle factors compared with women in the general population. For example, a short sleep duration and night shift work, which showed significant associations with dysmenorrhea, are common among working nurses.^32^ Second, many participants worked as clinical nurses who were unlikely to have serious dysmenorrhea that necessitated bed rest. This might cause selection bias toward healthy workers. However, the prevalence of dysmenorrhea (grades 2, 3, and 4) in the present study was so close to the prevalence in the general Japanese population that the potential bias seemed to have little effect on the prevalence of the three grades of dysmenorrhea. Third, this study was based on a cross-sectional analysis and, therefore, cannot determine whether the identified variables are risk factors or consequences of dysmenorrhea; however, this may be difficult to determine even in longitudinal studies.

## Conclusions

The JNHS (*n* = 36,665) revealed that the prevalence of dysmenorrhea was negatively associated with age. Furthermore, smoking increased the risk of dysmenorrhea by 20%, and this prevalence was not decreased to the original level during the 5 years since quitting smoking. In contrast, soybean intake with tofu reduced the risk of dysmenorrhea in a dose-dependent manner, which might be related to the production of PGs. Further research is necessary to confirm and expand upon these findings. Lastly, there were some participants with missing lifestyle variable data (*i.e.*, the “unknown” group), which might have introduced potential bias into the results. However, the sensitivity analysis in which the PRs were recalculated after excluding the “unknown” group obtained similar results to the primary results, which suggests that the missing data were unlikely to have introduced significant bias.

## Abbreviations Used

BMI: body mass index
CI: confidence interval
JNHS: Japan Nurses’ Health Study
PG: prostaglandin
PGE2: prostaglandin E2
PR: prevalence ratio
QOL: quality of life
