# Supplementary material for: Associations Between Lifestyle Factors and Primary Dysmenorrhea in the Japan Nurses’ Health Study
Source: Womens Health Rep (New Rochelle). 2025 Jul 23;6(1):702–10. doi: 10.1177/26884844251362183 (PMC12506579; doi:10.1177/26884844251362183)
Supplement: Supplementary Table S1 [file 26884844251362183_supp_tables1.pdf]

Supplemental Material

Table S1. Prevalence Ratios of Reproductive and Lifestyle Factors

		Age-adjusted RR [1]				Multivariable-adjusted PR [2]			
		PR	95% CI			PR	95% CI		
Age (years)	20–29					ref			
	30–34					0.86	0.79	−	0.94
	35–39					0.76	0.70	−	0.84
	40–44					0.65	0.59	−	0.71
	45–49					0.47	0.42	−	0.53
Current menstrual cycle	≤25 days	1.01	0.96	−	1.06	1.02	0.96	−	1.09
	26–31 days	ref				ref			
	32–50 days	0.97	0.92	−	1.01	0.96	0.90	−	1.02
	Irregular	1.17	1.13	−	1.21	1.13	1.08	−	1.19
Marital status	Single	ref				ref			
	Married [3]	0.77	0.74	−	0.79	1.04	0.98	−	1.10
Parity	None	ref				ref			
	One or more	0.69	0.67	−	0.71	0.69	0.65	−	0.72
BMI	<18.5	1.14	1.10	−	1.19	1.12	1.06	−	1.18
	18.5 to <25.0	ref				ref			
	25.0 to <30.0	1.02	0.97	−	1.08	1.02	0.95	−	1.09
	≥30.0	1.06	0.95	−	1.18	0.93	0.80	−	1.07
Smoking status	Never smoked	ref				ref			
	Current smoker	1.27	1.23	−	1.32	1.18	1.13	−	1.23
	Ex-smoker: < 5 years since quitting	1.14	1.07	−	1.22	1.15	1.05	−	1.25
	Ex-smoker: ≥ 5 years since quitting	1.14	1.08	−	1.20	1.10	1.03	−	1.19
	Ex-smoker: unknown duration	1.06	0.85	−	1.33	1.00	0.69	−	1.45
Alcohol consumption	None	ref				ref			
	≤2 days per week	1.10	1.06	−	1.14	1.05	1.00	−	1.10
	≥3 days per week	1.19	1.15	−	1.24	1.14	1.09	−	1.21
Engaging in nightshift	No	ref				ref			
	Yes	1.12	1.07	−	1.17	1.07	1.02	−	1.13
Moderate or vigorous physical activity [4] (≥10 MET hours/week)	No	ref				ref			
	Yes	1.13	1.06	−	1.21	1.13	1.05	−	1.21
Sleep duration	<5 hours/day	1.46	1.34	−	1.58	1.35	1.21	−	1.51
	5 hours/day	1.25	1.19	−	1.30	1.21	1.14	−	1.28
	6 hours/day	1.12	1.08	−	1.16	1.12	1.07	−	1.17
	7 hours/day	ref				ref			
	8 hours/day	0.93	0.88	−	0.98	0.99	0.92	−	1.06
	≥9 hours/day	1.09	0.95	−	1.25	1.04	0.87	−	1.24
Total isoflavone aglycone equivalents (mg/week)	Q1	ref				ref			
	Q2	0.94	0.90	−	0.99	1.02	0.96	−	1.08
	Q3	0.92	0.88	−	0.96	1.00	0.95	−	1.06
	Q4	0.91	0.87	−	0.95	1.00	0.94	−	1.06
	Q5	0.88	0.84	−	0.92	0.98	0.92	−	1.04

1) RR: Prevalence ratio

2) Adjusted for age, current menstrual cycle, parity, marital status, BMI, smoking status, alcohol consumption, engaging in nightshift, physical activity, sleep duration, and isoflavone intake

3) Includes divorced and widowed

4) Moderate to vigorous physical activity: intensity ≥3 METs
