# Supplementary material for: Associations Between Lifestyle Factors and Primary Dysmenorrhea in the Japan Nurses’ Health Study
Source: Womens Health Rep (New Rochelle). 2025 Jul 23;6(1):702–10. doi: 10.1177/26884844251362183 (PMC12506579; doi:10.1177/26884844251362183)
Supplement: Supplementary Table S2 [file 26884844251362183_supp_tables2.pdf]

Supplemental Material

Table S2. Dysmenorrhea and the Frequency of Soybean Consumption

		Multivariable-adjusted[1)]		
		PR[2)]	95% CI	Linear trend
Tofu	Never	ref		
	Once a week	0.96	0.89 - 1.04	
	2–3 days a week	0.94	0.87 - 1.02	p = 0.0555
	4–5 days a week	0.95	0.87 - 1.04	
	Almost every day	0.88	0.80 - 0.98	
Miso soup	Never	ref		
	Once a week	0.97	0.90 - 1.05	
	2–3 days a week	1.02	0.94 - 1.09	p = 0.4242
	4–5 days a week	1.00	0.92 - 1.09	
	Almost every day	1.01	0.94 - 1.09	
Natto	Never	ref		
	Once a week	0.99	0.95 - 1.03	
	2–3 days a week	1.01	0.96 - 1.07	p = 0.9205
	4–5 days a week	0.92	0.85 - 1.00	
	Almost every day	1.05	0.96 - 1.15	

1) Adjusted for age, current menstrual cycle, parity, marital status, BMI, smoking status, alcohol consumption, engaging in nightshift, physical activity, and sleep duration

2) PR: prevalence ratio
